# Pyloric Metrics and Distensibility Plateau by Functional Luminal Imaging Probe in Pediatric Patients: A Pilot Study

**DOI:** 10.1111/nmo.70303

**Published:** 2026-04-02

**Authors:** Peter T. Osgood, Natalie V. Hoffmann, Joshua B. Wechsler, Roman Pantazopoulos, John E. Fortunato

**Affiliations:** ^1^ Division of Pediatric Gastroenterology, Hepatology, and Nutrition Ann & Robert H. Lurie Children's Hospital of Chicago Chicago Illinois USA; ^2^ Department of Pediatrics Northwestern University Feinberg School of Medicine Chicago Illinois USA

**Keywords:** dyspepsia, functional luminal imaging probe (FLIP), gastroparesis, nausea, pylorus

## Abstract

**Background & Aims:**

Pyloric dysfunction contributes to chronic nausea, vomiting, and abdominal pain in adults and children. The Functional Luminal Imaging Probe (FLIP) has been used in adults to characterize pyloric distensibility as a marker of muscular hypertonicity and to predict symptomatic improvement to intrapyloric botulinum toxin injection (IPBI). FLIP parameters that identify pyloric dysfunction in children are unknown. In this retrospective cohort study, we aimed to characterize the relationship of pyloric FLIP parameters to clinical symptoms, gastric emptying (GE), and antroduodenal manometry (ADM) findings in pediatric patients. Secondarily, we aimed to assess FLIP metrics as related to the subjective response to IPBI.

**Methods:**

Chart review was performed for patients undergoing pyloric FLIP for refractory upper gastrointestinal symptoms at Lurie Children's Hospital. FLIP parameters were compared to symptoms, demographics, medical history, GE, ADM findings, and documented response to IPBI. The presence of a distensibility plateau (DP) was assessed.

**Results:**

Ninety‐nine patients underwent pyloric FLIP and 74 were treated with IPBI. Pyloric distensibility index (pDI) correlated with age and biological gender. Patients with chronic nausea, early satiety, concurrent pain disorders, joint hypermobility, and orthostatic intolerance demonstrated higher pDI compared to other symptoms. While pDI did not correlate with GE, it was lower in patients with normal fasting patterns on ADM and higher in IPBI responders. Nearly half of patients demonstrated a DP for which pDI was significantly higher in IPBI responders.

**Conclusions:**

This pilot study supports pyloric dysfunction as a mechanism underlying chronic upper gastrointestinal symptoms and would support prospective investigation of pyloric FLIP metrics in pediatrics.

## Key Points

- The functional luminal imaging probe (FLIP) has been used to characterize pyloric distensibility as related to gastric emptying, upper gastrointestinal (GI) symptoms, and clinical response to pyloric inhibition with botulinum toxin A in adults, but the utility of these metrics is poorly defined in children and adolescents.
- Our retrospective pediatric cohort study demonstrated higher pyloric distensibility in female patients and older patients. Pyloric distensibility was not predictive of gastric emptying and patients with higher pyloric distensibility were more likely to report improvement following botulinum toxin therapy, contrary to adult studies.
- This study is the first to report a distensibility plateau (DP) effect at the pylorus. The presence of a DP enhanced the ability to predict response to botulinum toxin, suggesting that the DP may become a clinically useful metric in pediatric patients with chronic upper GI symptoms.

## Graphical abstract

Pediatric patients underwent pyloric functional luminal imaging probe (FLIP) for refractory upper gastrointestinal symptoms. Unlike prior adult studies, pyloric distensibility did not correlate to gastric emptying, and patients with higher distensibility more frequently responded to botulinum toxin. The presence of a distensibility plateau enhanced the observed response to botulinum toxin.

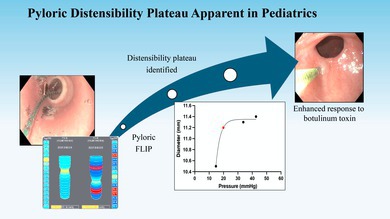

## 1 Introduction

Chronic nausea, vomiting, and abdominal pain are challenging to classify and treat in the pediatric population due to a paucity of objective biomarkers and limited treatment options [1, 2, 3]. Refractory symptoms can have a substantial impact on growth and development, mental health, and quality of life and contribute to a substantial health care burden [4, 5, 6, 7]. Medication and procedural interventions are often offered empirically due to lack of clinical phenotyping tools and limited mechanistic testing to effectively guide therapeutic options.

Pyloric dysfunction is an established pathophysiologic process underlying chronic nausea and vomiting [8]. Pyloric‐directed therapies for refractory gastroparesis, including intrapyloric botulinum toxin injection (IPBI), pyloric dilation, and gastric peroral endoscopic myotomy (G‐POEM), target pyloric nonrelaxation and have demonstrated success in adults [9, 10, 11, 12, 13, 14, 15, 16, 17, 18, 19, 20, 21]. Pediatric studies are limited [3, 22, 23, 24, 25]. Two groups have recently reported significant improvement in patient‐reported nausea and vomiting in pediatric patients after IPBI [3, 24]. However, predictors of response to IPBI are lacking, limiting effective patient selection.

Functional Luminal Imaging Probe (FLIP) has been effectively utilized to characterize esophageal distensibility, which is characterized by a distensibility plateau (DP) [26, 27, 28]. Pyloric distensibility measured by FLIP has been used to assess pyloric sphincter characteristics and guide therapeutic measures with varying success. In adults, pyloric distensibility predicted clinical benefit to both IPBI and G‐POEM [13, 16]. Hirsh et al. described a cohort of children treated with IPBI in which pyloric distensibility was assessed with FLIP [3]. No pediatric study to date has defined FLIP parameters to objectively diagnose pyloric pathology or predict success of IPBI or other interventions. Furthermore, it is unknown if the pylorus has a DP.

In this single‐center observational cohort study, we aimed to explore the role of pyloric dysfunction in patients with chronic nausea, pain, and vomiting. We assessed the utility of pyloric distensibility as a meaningful clinical phenotyping tool to guide pyloric‐directed therapies. We hypothesized that pyloric distensibility predicts the response to IPBI.

## 2 Materials and Methods

This observational cohort study included patients followed at a single tertiary care pediatric center who underwent pyloric FLIP from 11/2020 through 12/2022 to evaluate symptoms of chronic nausea, vomiting, abdominal pain, early satiety, distention, and abnormal weight trajectory refractory to standard medical therapies. Clinical, endoscopic, FLIP, and manometry data were obtained via chart review. The study was approved by the Ann & Robert H. Lurie Children's Hospital of Chicago Institutional Review Board. Patients were excluded if peak intraballoon pressure was < 15 mmHg, per manufacturer guidelines [29].

### 2.1 Clinical, Endoscopic, and Manometric Assessment

Pre‐operative gastric symptoms (vomiting, nausea, abdominal pain, early satiety, distension/bloating, and poor weight gain/weight loss) were obtained from provider documentation at the clinic visit(s) preceding the pyloric FLIP procedure. All patients underwent standard‐of‐care endoscopy and pyloric FLIP. Among patients treated with IPBI, clinical response was obtained from provider documentation at the clinic visit following the pyloric FLIP and IPBI procedure. Response to IPBI was defined as patient or caregiver report of partial to complete improvement in at least one preoperative symptom. Post hoc assessment of response was reliant on patient recall and provider documentation and could thus only be graded on a binomial basis of response or nonresponse. Objective symptom severity measures could not be captured due to these limitations. Endoscopic biopsies were examined by gastrointestinal (GI) pathologists. Histologic findings were defined as follows: reflux esophagitis included < 15 eosinophils per high power field (eos/hpf) in esophageal biopsies, acute esophagitis included neutrophilic esophageal inflammation, eosinophilic esophagitis included ≥ 15 eos/hpf in esophageal biopsies, chronic active gastritis included neutrophilic infiltration of gastric mucosa, peptic duodenitis included active inflammation with gastrin mucin‐cell metaplasia and Brunner gland hyperplasia. Histologic findings of inactive chronic gastritis or reactive gastropathy were not included in abnormal histologic findings. Gastric emptying was assessed for patients who underwent four‐hour solid‐meal gastric emptying scintigraphy (GES). Delayed gastric emptying was interpreted based on consensus recommendations [30]. Patients did not routinely have gastric emptying reassessed post‐IPBI. Antroduodenal manometry (ADM) data were included if available. ADM studies were subject to variable protocols dependent on clinical circumstances, catheter location, or provider preference. ADM interpretation included distal‐most antral contraction amplitude [31], presence of fasting Phase III migrating motor complexes (MMCs), and post‐prandial antral hypomotility (PPAHM) [25]. The distal‐most antral wave as an indirect surrogate measure of pyloric contraction was chosen since isolation of pyloric waveforms on ADM is difficult owing to a short zone of muscular activity, movement of the ADM catheter, and difficulty identifying the pylorus among other waveforms [24, 31, 32, 33]. Pylorospasm was defined as sustained tonic or phasic contractions (≥ 10 mmHg / ≥ 3 min) of the most distal antral sensor [34, 35].

### 2.2 Pyloric Distensibility Assessment Using FLIP

FLIP studies were performed using the Endoflip 2.0 Impedance Planimetry System (Endoflip EF‐200; Medtronic Inc., Minneapolis, MN) with an 8‐cm catheter (Endoflip EF–325 N). After standard endoscopy, a FLIP catheter was calibrated to atmospheric pressure and placed transorally or via gastrostomy into the stomach and across the pylorus under endoscopic guidance. Placement was confirmed by appearance of a waist at the central sensors during balloon inflation. Stepwise 5–10 mL distensions were performed, beginning at 20 mL and increasing to a maximum of 50 mL. Measurements were taken between 15 and 60 mmHg intraballoon pressure, per manufacturer guidelines [29]. Each distension was maintained for 30 s to allow catheter equilibration. At each distension volume (ml), the following were recorded: pressure (mmHg), diameter (mm) at the narrowest luminal area, and distensibility index (DI, mm^2^/mmHg). DI is calculated by dividing the cross‐sectional area by pressure. Peak pyloric DI (pDI) and diameter were utilized for analysis. To assess for DP, we compared pyloric diameter to balloon pressure for each patient and applied a nonlinear exponential plateau model using GraphPad Prism v9.3.1. All patients were under general anesthesia with propofol during FLIP without use of sevoflurane, opioids, or neuromuscular blockade [3].

### 2.3 Intrapyloric Botulinum Toxin Injection

Recommendation for pyloric‐directed therapy was at the discretion of the treating provider with consideration of pyloric distensibility and clinical impact. IPBI was administered by one of three endoscopists following FLIP measurements. Botulinum toxin was reconstituted in sterile saline just prior to injection, per manufacturer guidelines (*Allergan*), to a concentration of 50 IU/mL. The pylorus was identified visually during endoscopy and injected in four quadrants via sclerotherapy needle to a total dose of 100 IU botulinum toxin.

### 2.4 Statistical Analysis

Statistical analysis was performed with R v4.4.1 and GraphPad Prism v9.3.1. Descriptive statistics for continuous variables were reported as mean with standard deviation. Groups were compared using chi‐squared test for categorical variables. For group comparison of pDI, analysis of covariance (ANCOVA) was used on log transformed pDI (for normalization). The Shapiro test was used to assess for normalized distribution. Spearman (ρ) was calculated using *pcor.test* to determine correlation of pDI with ADM measures with control for age/gender. *p* value < 0.05 was considered statistically significant.

## 3 Results

### 3.1 Patient Characteristics

**FIGURE 1:**
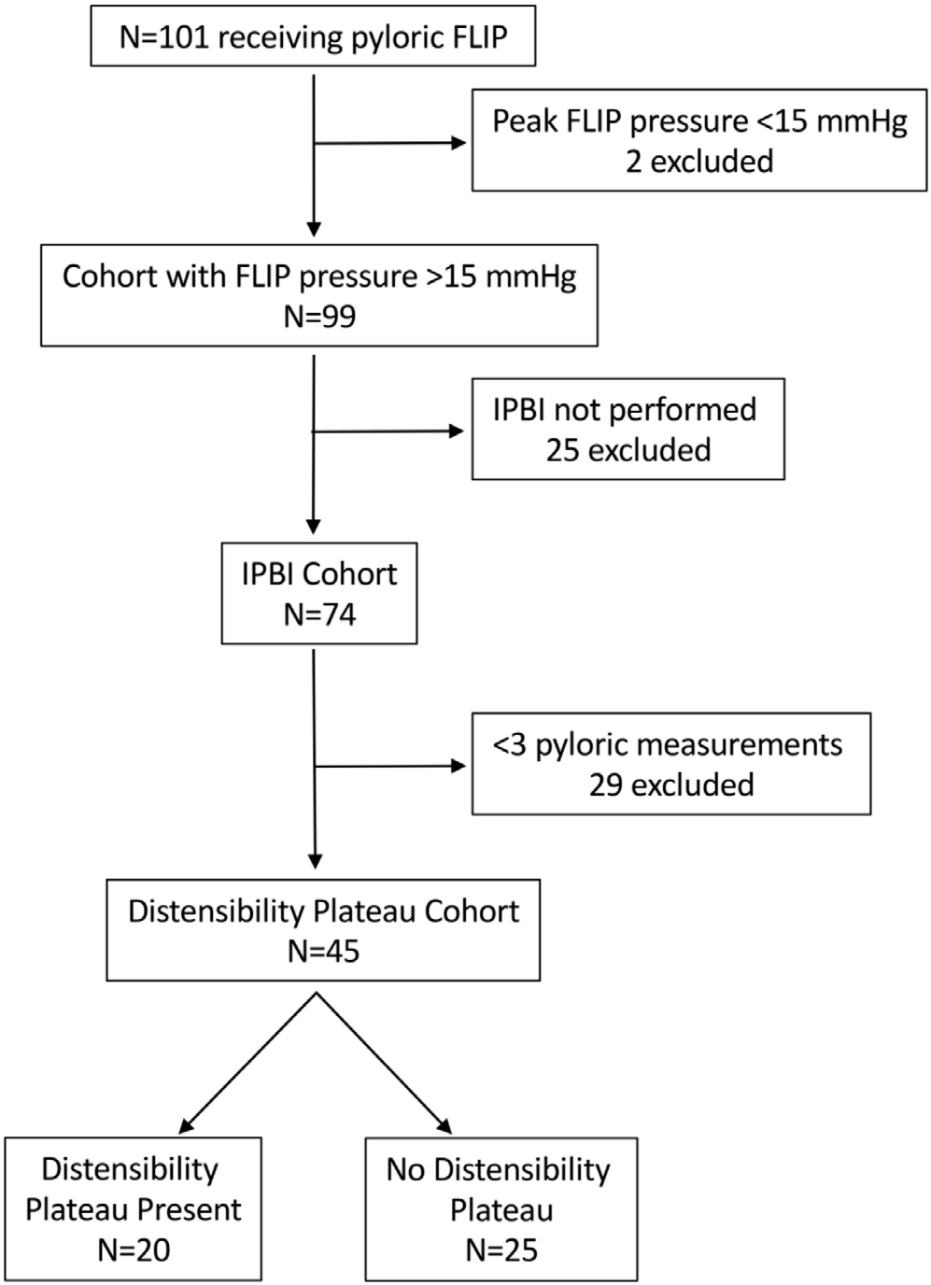
Consort diagram.

One hundred one patients underwent pyloric FLIP during our study period. Two patients were excluded due to pressure < 15 mmHg in the FLIP balloon (Figure 1). Patient characteristics are summarized in Table 1. Within the final cohort, mean age was 14 years with a female predominance (73%). Most patients identified as white race (71%). Predominating symptoms prior to FLIP included abdominal pain (78%), nausea (74%), distension/bloating (69%), early satiety (60%), vomiting (54%), and poor weight gain or weight loss (49%). Medical history was notable for a history of feeding support (42%), chronic pain (62%), anxiety/depression (58%), concurrent presence of irritable bowel syndrome (IBS, 37%), Ehlers‐Danlos syndrome/hypermobility (41%), and orthostatic intolerance (OI)/postural orthostatic tachycardia syndrome (POTS) (66%). Among the patients, 25% had abnormal biopsies of the esophagus, stomach, or duodenum. No patients had pyloroplasty or antireflux surgery performed prior to FLIP or IPBI. A majority of patients (88%) had contrast imaging to verify normal nonobstructive anatomy (Table 1).

**TABLE 1:** Patient characteristics prior to FLIP.

Characteristic	Overall (*N* = 99)^a^
Demographics	
Age (years)	14 ± 6
Gender (female)	72 (74)
Race/Ethnicity	
White	70 (71)
Black	8 (8)
Asian	3 (3)
Other	18 (18)
Hispanic/Latinx	12 (12)
Growth Parameters	
Weight Z‐score	−0.3 ± 1.3
BMI Z‐score	−0.1 ± 1.3
Symptoms Prior to FLIP	
Vomiting	53 (54)
Nausea	73 (74)
Abdominal Pain	77 (78)
Early Satiety	59 (60)
Distension/Bloating	68 (69)
Poor Weight Gain/Weight Loss	48 (49)
Medical History	
Feeding Support	42 (42)
Cyclic Vomiting syndrome	4 (4)
Chronic pain	61 (62)
Upper GI Series Performed	84 (85)
Abnormal GES^b^	25 (43)
Irritable Bowel syndrome (IBS)	36 (37)
Anxiety/Depression	57 (58)
Ehlers‐Danlos/hypermobility	41 (41)
POTS/Orthostatic Intolerance	65 (66)
Abnormal biopsies^c^	24 (25)

^a^ For continuous variable, display is mean ± SD. For categorical variables, display is N (%).

^b^ 58 patients had a 4‐h solid phase gastric emptying study (GES).

^c^ 95 of 99 patients had biopsies. Abnormalities included eosinophilic esophagitis, ganglioneuroma, *H. pylori* gastritis, intra‐epithelial lymphocytes in the duodenum, peptic duodenitis, reflux esophagitis, reflux esophagitis + gastritis.

### 3.2 Distensibility Index and Patient Characteristics

We initially assessed the relationship of patient characteristics to pDI. We found a significant correlation of pDI with age (ρ = 0.53, *p* < 0.001) and increased pDI in females (*p* = 0.01) (Figure S1A,B). We therefore performed ANCOVA or partial correlation for the remaining comparisons to control for age/gender. For symptoms (Figure S2A), increased pDI was identified in patients with nausea (7 ± 5 vs. 5 ± 2, *p* < 0.001) and early satiety (6 ± 5 vs. 9 ± 5, p < 0.001). For medical history (Figure S2B), we observed decreased pDI in patients receiving feeding support (*p* < 0.01), but increased pDI in patients with IBS (*p* = 0.038), chronic pain (*p* < 0.001), anxiety/depression (p < 0.001), Ehlers‐Danlos syndrome (EDS)/hypermobility (*p* = 0.04), and POTS/OI/Dysautonomia (*p* < 0.001).

### 3.3 Gastric Functional Characterization and pDI

We next assessed the association of pDI with gastric emptying and ADM parameters (Figure S3). We found no differences based on delayed gastric emptying. We found decreased pDI in patients with normal fasting patterns on ADM (*p* = 0.001), but increased pDI in patients with neuropathic findings (*p* < 0.001). There was no correlation of pDI to peak antral pressures or the duration of pylorus spasm.

### 3.4 Patient Characteristics by IPBI Response

We next assessed our cohort based on IPBI response (*N* = 74), excluding 25 patients in whom IPBI was not performed (Figure 1). We examined differences in patient characteristics based on symptomatic response to IPBI. We did not identify differences in IPBI response based on demographics, symptoms prior to FLIP, medical history, or gastric emptying (Table S1).

### 3.5 Gastric Functional Characterization and IPBI Response

**FIGURE 2:**
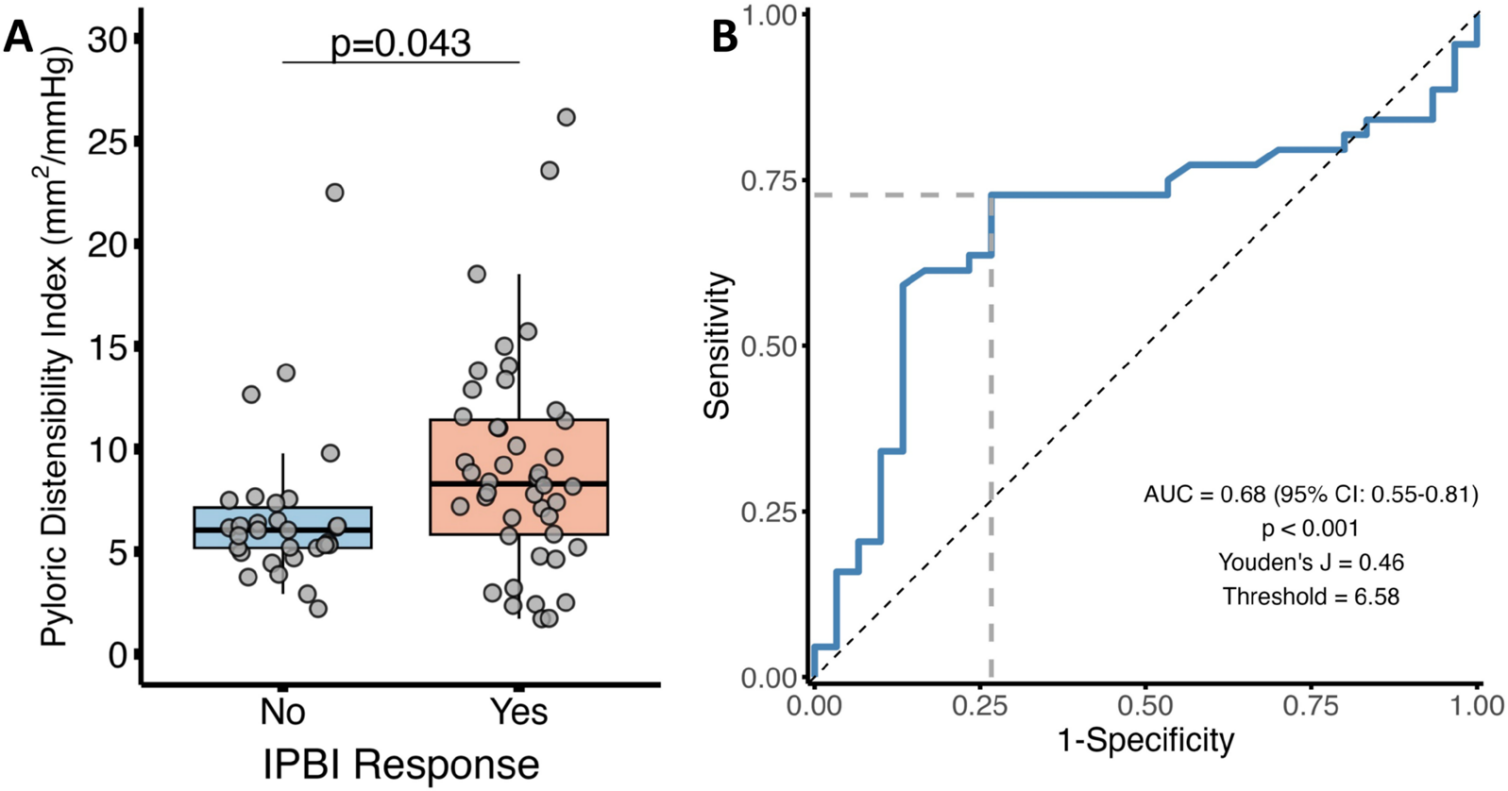
Pyloric Distensibility Index (pDI) is increased with symptomatic improvements from IPBI and predicts the response. Comparison of pDI by IPBI response with ANCOVA to control for age/gender (A). ROC Curve for pDI to predict IPBI response (B) with dashed gray line indicating optimal threshold.

We next assessed for differences in pDI and gastric motility measures based on the response to IPBI. We found significantly higher pDI among patients who responded to IPBI (Figure 2A). We further assessed the ability of pDI to predict IPBI response via receiver operating characteristics (ROC, Figure 2B), which demonstrated moderate discrimination (AUC 0.68, *p* < 0.001). We observed a higher incidence of pylorus spasm ≥ 3 min among patients without a response to IPBI, most commonly following stimulation with a motilin agonist, but otherwise no differences were noted (Figure S4).

### 3.6 Two Distinct Distensibility Patterns

**FIGURE 3:**
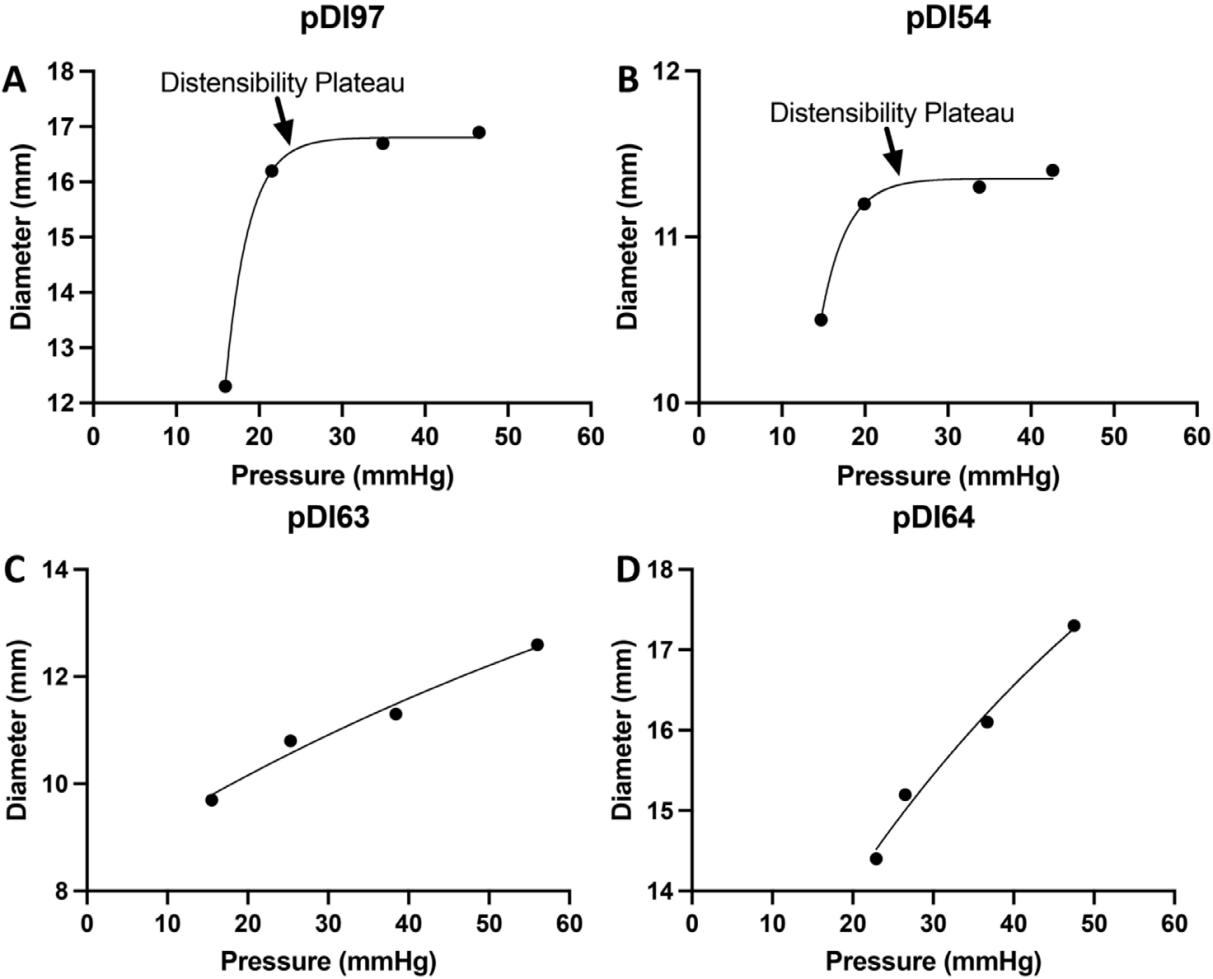
Representative graphs for a distensibility plateau (DP). The DP was identified by comparing diameter and pressure with a nonlinear exponential plateau model. Individual patient graphs shown with a DP (A, B) vs. no DP (C, D).

In adults, normal pDI is considered > 10 mm^2^/mmHg [16, 36]. Among our cohort, pDI was predominantly < 10 mm^2^/mmHg with a non‐normalized distribution (Figure S5A,B) despite pyloric diameter, volume, and pressure demonstrating a normal distribution (Figure S5C–H). To better understand the functional phenotypes associated with the pylorus, we assessed for a DP. To do this, we assessed patients with at least three serial FLIP measurements (*n* = 45, Figure 1) and compared pyloric diameter to balloon pressure for each patient using a nonlinear exponential plateau model. This highlighted a DP in 20 patients, but 25 without a DP (Figure 3). No difference was seen in pDI based on the presence of a DP (data not shown).

### 3.7 IPBI Response and DP

**FIGURE 4:**
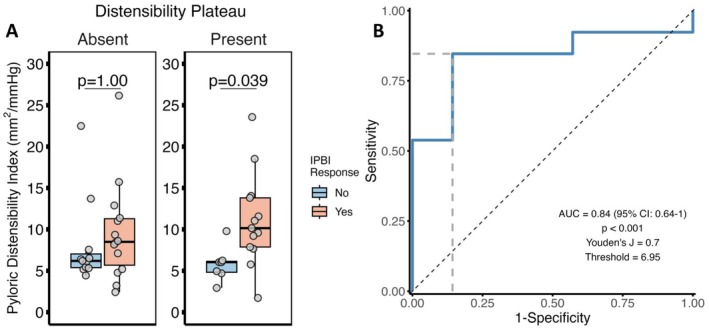
Differences and predictive utility of pyloric distensibility index (pDI) in IPBI responders is enhanced with distensibility plateau (DP) identification. Comparisons of pDI by ANCOVA to control for age/gender stratified by IPBI response and DP identification (A). *p* values shown were post hoc adjusted by Bonferroni. ROC Curve for pDI to predict IPBI response in patients with a DP (B).

We lastly assessed the extent to which pDI differences existed among patients based on IPBI response stratified by the presence of a DP. After controlling for age/gender, we found that patients with a DP had significantly increased pDI among IPBI responders (*p* = 0.039, Figure 4A), while patients without a DP did not (*p* = 1.0). We generated a ROC curve to assess the extent to which pDI predicted IPBI response in patients with a DP (Figure 4B) and we observed an AUC of 0.84 (*p* < 0.001).

## 4 Discussion

The pyloric sphincter has an established role in gastric emptying [8, 37]. Pyloric dysfunction, characterized by manometry and FLIP, demonstrates elevated basal tone, the presence of pylorospasm, and alterations in distensibility and diameter, which correlate with impaired GE and upper GI symptoms [13, 24, 34, 38, 39]. However, the utility of pyloric FLIP to guide symptom assessment and therapy decisions remains unclear. In this study, we aimed to better understand the pathophysiology of the pylorus as related to chronic nausea, vomiting, early satiety, abdominal pain, distention, inadequate weight gain, and delayed gastric emptying in pediatrics, as well as response to IPBI. Congruent with prior data [24], manometry data from our cohort supported pyloric nonrelaxation, rather than aperistalsis, as a mechanism of symptoms and delayed GE in pediatrics. While impaired pyloric relaxation is also often demonstrated in adults, impaired post‐prandial antral contractions may also contribute to delayed GE and symptomatology [40], a finding not observed within our pediatric cohort.

Here we focused on the relationship of pDI to clinical characteristics, gastric functional measures, and IPBI response. Given variation in patient size within a pediatric cohort, we anticipated that volume‐based measurements would be unreliable and, rather, utilized peak pDI and peak pyloric diameter for our assessment. We identified an association of pDI with age, gender, symptoms of early satiety, nausea and vomiting, as well as a medical history of anxiety/depression, chronic pain, EDS, feeding support, and OI/POTS. In addition, we observed higher pDI among IPBI responders and importantly identified two phenotypes of pyloric distensibility based on the presence of a DP. This DP enhanced the ability to predict IPBI response with pDI. Together, this informs the role of pyloric distensibility in chronic upper GI tract symptoms and implicates a role for measurement of pDI in management.

Our understanding of the pediatric pylorus is limited by lack of normative data, particularly with respect to age, size, nutritional status, or underlying comorbidities. We identified a significant correlation of pDI with age and gender, suggesting the need for normative values, potentially stratified by patient characteristics. Adult studies have suggested that a pDI > 10 mm^2^/mmHg is normal [13, 41] and that pDI below this cutoff is correlated to poor GE and greater response to IPBI^17^. Our study, however, would contradict these previous conclusions, a finding that was both counterintuitive and unexpected. The pDI cutoff value of 10 mm^2^/mmHg was not applicable to our cohort nor prior published pediatric cohorts [3, 42] whereby pDI is frequently < 10. Though a clear numeric cutoff for normal versus abnormal pDI was not apparent in our study, our work suggests that optimal candidates for IPBI may be identified by relatively higher pDI values, particularly in the presence of a DP. These findings are supported by a study in which greater baseline pyloric compliance and distensibility correlated with symptomatic improvement following IPBI^36^. These contradictory findings, however, underscore the need to define normative pyloric FLIP parameters and patient phenotypes in pediatrics and may suggest variable pathophysiology in pediatric patients when compared to adults.

A critical observation was the association of specific symptoms with pDI, including increased pDI with early satiety and nausea along with decreased pDI with vomiting. The role of the pylorus in specific GI symptoms in pediatric patients is unclear. Our data suggest that vomiting may be secondary to more highly inhibited pyloric relaxation compared to other upper GI symptoms. This observation may suggest differing pathophysiology underlying these observed symptoms.

Botulinum toxin A produces a transient period of functional paralysis of the pyloric sphincter and is increasingly used for symptoms of refractory nausea and vomiting in children [3, 23, 24, 25, 42, 43, 44]. Just as pDI correlated to reported symptoms, the presence of two phenotypes based on DP suggests differing symptom pathogenesis. Without a control population, it is unclear which pattern of distensibility (DP or no DP) is more pathologic in nature. Within our cohort, however, the greater response observed to IPBI in those with a DP may suggest that this pattern signifies a specific pathologic state. We postulate that the presence of a DP indicates a distinct pyloric muscle pathology, whereby limitations in pyloric compliance create an abrupt ceiling effect observed as the DP. Since those with a DP and higher degrees of pDI were more likely to respond to IPBI, this may suggest that lower pDI stems from alternative pathology such as fibrosis or hypertonicity in excess of that which might be overcome by IPBI^45^. Lack of a DP is also a critical finding suggesting that pathology outside of the pylorus may be driving symptoms. In these cases, we must consider alternative pathology including depletion of the interstitial cells of Cajal [37, 45] or dysregulated neuroimmune signaling [46, 47]. Further research is needed to validate this hypothesis, but characterization of the DP may be instrumental in phenotyping patients to identify optimal selection for IPBI or other therapies such as pyloric balloon dilatation, prokinetics, or neuromodulation. Validation in prospective, randomized studies and investigations of treatments such as dilatation are necessary.

Our study had several notable strengths. We had a large cohort of patients who were evaluated in a nearly standardized manner. Our cohort was diverse in ages and included those with normal and delayed GE. We controlled for age throughout the analysis, strengthening each observation. The analysis of patients with ≥ 3 FLIP measurements allowed us to illustrate the pattern of a DP for some versus no DP for others.

Our study was limited by its retrospective nature, provider discretion for use of IPBI, lack of a standardized FLIP protocol and validated symptom scoring measures, and variable follow‐up timeframes. Though we kept anesthesia protocols standardized for all patients undergoing FLIP, it is also very possible that the use of anesthesia such as propofol could falsely lower pyloric distensibility within our cohort [48], making comparison to adult norms difficult. As outcomes are defined by patient‐reported symptoms and reliant on provider documentation, the true magnitude of effect from IPBI is debatable and the potential for placebo effect exists [9, 15, 49]. We were unable to analyze IPBI response in the context of specific symptoms given the variable prevalence within the IPBI cohort.

Overall, this study served as a pilot investigation into the role of pyloric FLIP metrics as related to patient characteristics, GI symptoms, gastric function, and response to a sometimes‐controversial therapy. The findings of this study are still far from generalizable to routine medical practice. Our goal was to aid in the design of future studies, improve mechanistic understanding, and guide potential therapeutic options. Further studies should interrogate the relationship of GE, clinical symptoms, and response to IPBI as related to pDI and DP. This would be more generalizable with prospective data collection, validated symptom measures, a standardized FLIP protocol, and a standardized timeframe.

## 5 Conclusion

In conclusion, our study suggests that pyloric FLIP plays a role in differentiating patients that may benefit from targeted pyloric therapies. It will be critical for multicenter collaboration and standardization of procedural technique and symptom assessment to reach an evidence‐based understanding of the role of the pylorus in chronic nausea, vomiting, and abdominal pain in pediatrics to establish the most suitable treatments.

## Author Contributions

Peter T. Osgood, review of data, primary author of submitted manuscript. Natalie V. Hoffmann, first collection and analysis of data, first draft of manuscript. Joshua B. Wechsler, responsible for data analysis and critical review and revision of the manuscript. Roman Pantazopoulos, responsible for data collection and revision of the manuscript. John E. Fortunato, responsible for ensuring integrity of the data and critical review of the manuscript.

## Funding

The authors have nothing to report.

## Conflicts of Interest

The authors declare no conflicts of interest.

## Supporting information


**Figure S1:** Correlation of pyloric distensibility index with Age and Gender. Age correlated with peak pyloric distensibility index (A). Calculation of Spearman correlation coefficient with *p* < 0.05 considered significant. Increased pyloric distensibility index in females (B). Comparison by ANCOVA with control for age.
**Figure S2:** Differences in pDI by Symptoms and Medical History. (A) Increased pDI was seen in patients with early satiety and nausea. (B) Increased pDI was seen in patients with a history of anxiety/depression, chronic pain, EDS/hypermobility, and POTS/orthostasis while decreased pDI seen in patients requiring feeding support. Comparison by ANCOVA with Bonferroni post hoc *p* values shown after and adjusting for age/gender. *p* < 0.05 considered significant.
**Figure S3:** Association of pDI with gastric motility measures. (A) Decreased pDI was seen in patients with a neuropathic abnormality on ADM, while increased pDI was observed in patient with normal fasting response. N for each group shown above boxplot. Comparison by ANCOVA with Bonferroni post hoc *p* values shown after and adjusting for age/gender. (B) No correlation of pDI with peak antral pressures or duration of pylorospasm. Spearman (rho) correlation coefficient determined. N for each group shown to the right. *p* < 0.05 considered significant.
**Figure S4:** Association of IPBI response with gastric motility abnormalities. (A) Reduced prolonged pylorus spasm in patients that responded to IPBI. Comparison by Binary logistic regression. (B‐C) No differences in ADM measures or Duration of Pylorospasm based on IPBI response. Comparison by Mann–Whitney. *p* < 0.05 considered significant.
**Figure S5:** Distribution of pyloric FLIP parameters. Non‐normalized distribution observed for peak pyloric distensibility index (pDI) (A‐B) with most values < 10 mm2/mmHg. Normalized distribution of peak diameter (C‐D), and volume (E‐F) and pressure (G‐H) at the peak pDI.
**Table S1:** Patient Characteristics Based on IPBI Response.
1. For continuous variable, display is mean ± SD. For categorical variables, display is *n* (%). 2. Categorical variable: Chi‐Squared test, Normalized continuous variable: *t*‐test, non‐normalized continuous variable: Mann–Whitney. *p value < 0.05. 3. 52 patients had 4‐h solid phase gastric emptying study (GES). 4. Only 70 of 74 patients received biopsies at time of pyloric FLIP. Abnormal biopsies included: Eosinophilic Esophagitis, ganglioneuroma, *H. pylori* gastritis, Intra‐epithelial lymphocytes in the duodenum, peptic duodenitis, reflux esophagitis, reflux esophagitis + gastritis. 5. Measurements taken at peak distensibility index (DI). 6. Measurements taken at peak diameter.

## Data Availability

The data that support the findings of this study are available from the corresponding author upon reasonable request.
