# Supplementary material for: Pyloric Metrics and Distensibility Plateau by Functional Luminal Imaging Probe in Pediatric Patients: A Pilot Study
Source: Neurogastroenterol Motil. 2026 Apr 2;38(4):e70303. doi: 10.1111/nmo.70303 (PMC13044568; doi:10.1111/nmo.70303)
Supplement: Supplementary file 1 — Figure S1: Correlation of pyloric distensibility index with Age and Gender. Age correlated with peak pyloric distensibility index (A). Calculation of Spearman correlation coefficient with p < 0.05 considered significant. Increased pyloric distensibility index in females (B). Comparison by ANCOVA with control for age. Figure S2: Differences in pDI by Symptoms and Medical History. (A) Increased pDI was seen in patients with early satiety and nausea. (B) Increased pDI was seen in patients with a history of anxiety/depression, chronic pain, EDS/hypermobility, and POTS/orthostasis while decreased pDI seen in patients requiring feeding support. Comparison by ANCOVA with Bonferroni post hoc p values shown after and adjusting for age/gender. p < 0.05 considered significant. Figure S3: Association of pDI with gastric motility measures. (A) Decreased pDI was seen in patients with a neuropathic abnormality on ADM, while increased pDI was observed in patient with normal fasting response. N for each group shown above boxplot. Comparison by ANCOVA with Bonferroni post hoc p values shown after and adjusting for age/gender. (B) No correlation of pDI with peak antral pressures or duration of pylorospasm. Spearman (rho) correlation coefficient determined. N for each group shown to the right. p < 0.05 considered significant. Figure S4: Association of IPBI response with gastric motility abnormalities. (A) Reduced prolonged pylorus spasm in patients that responded to IPBI. Comparison by Binary logistic regression. (B‐C) No differences in ADM measures or Duration of Pylorospasm based on IPBI response. Comparison by Mann–Whitney. p < 0.05 considered significant. Figure S5: Distribution of pyloric FLIP parameters. Non‐normalized distribution observed for peak pyloric distensibility index (pDI) (A‐B) with most values < 10 mm2/mmHg. Normalized distribution of peak diameter (C‐D), and volume (E‐F) and pressure (G‐H) at the peak pDI. Table S1: Patient Characteristics Based on IPBI Response. 1. [file NMO-38-e70303-s001.pdf]

SUPPLEMENTAL MATERIALS

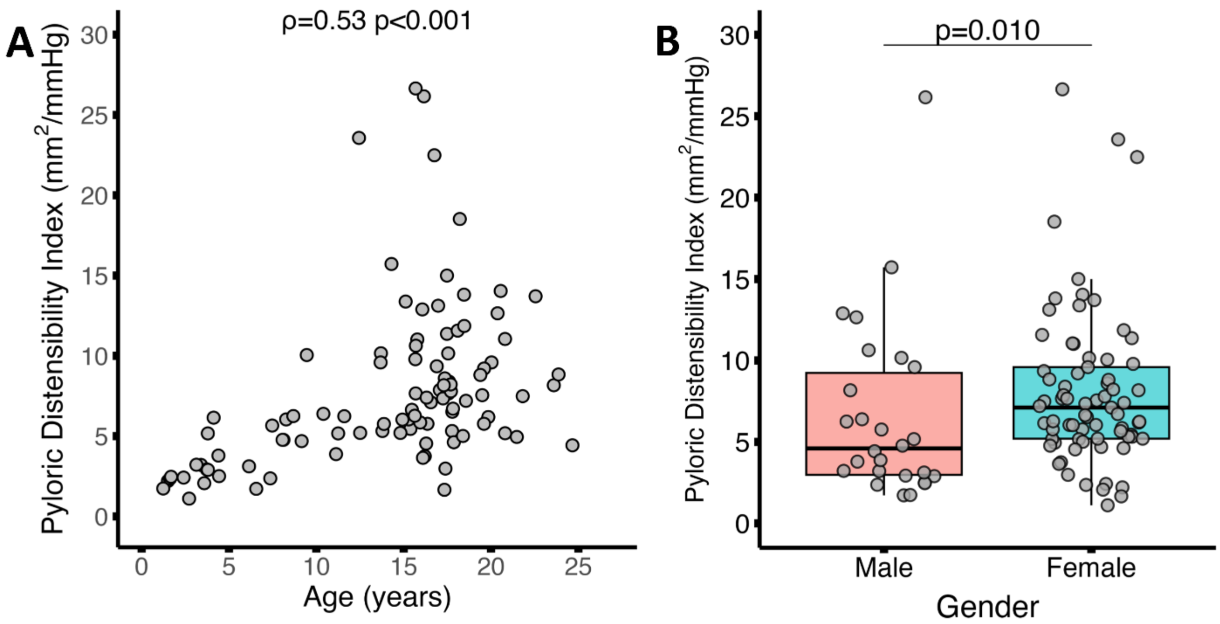

Supplemental Figure 1. Correlation of pyloric distensibility index with Age and Gender. Age correlated with peak pyloric distensibility index **(A)**. Calculation of Spearman correlation coefficient with $p<0.05$ considered significant. Increased pyloric distensibility index in females **(B)**. Comparison by ANCOVA with control for age.

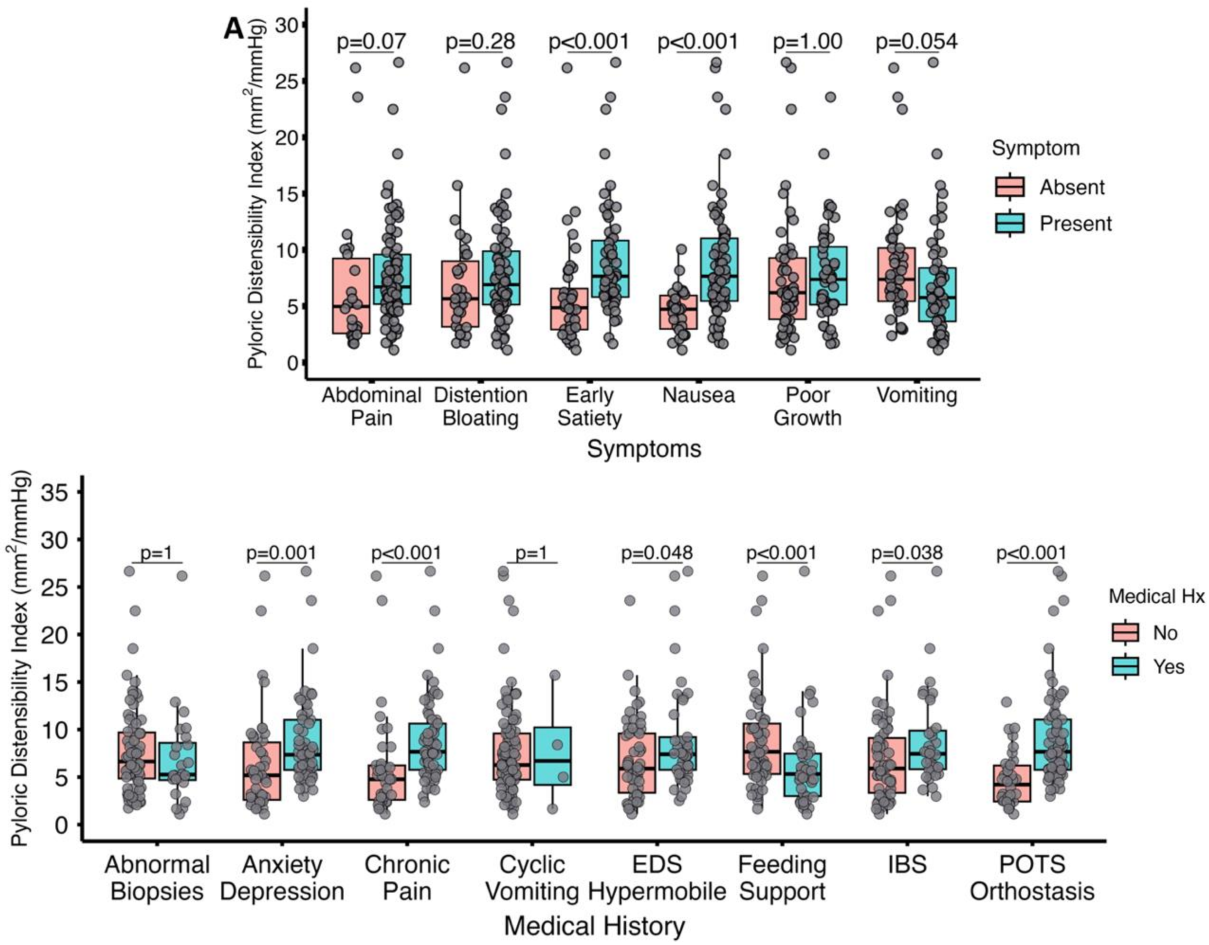

Supplemental Figure 2. Differences in pDI by Symptoms and Medical History. A) Increased pDI was seen in patients with early satiety and nausea. **B)** Increased pDI was seen in patients with a history of anxiety/depression, chronic pain, EDS/hypermobility, and POTS/orthostasis while decreased pDI seen in patients requiring feeding support. Comparison by ANCOVA with Bonferroni post-hoc p-values shown after and adjusting for age/gender. $p<0.05$ considered significant

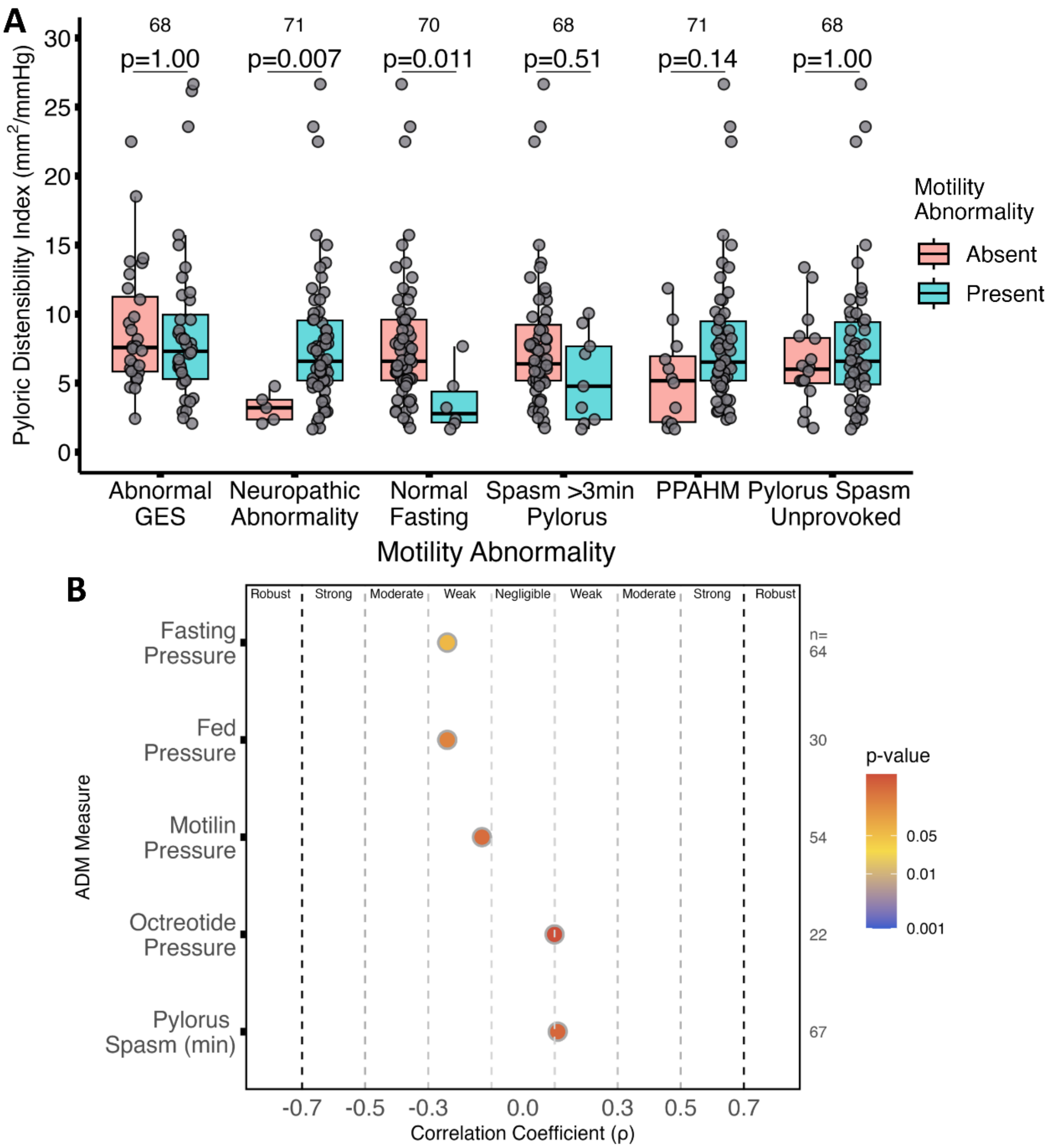

Supplemental Figure 3. Association of pDI with gastric motility measures. A) Decreased pDI was seen in patients with a neuropathic abnormality on ADM, while increased pDI was observed in patient with normal fasting response. N for each group shown above boxplot. Comparison by ANCOVA with Bonferroni post-hoc p-values shown after and adjusting for age/gender. **B)** No correlation of pDI with peak antral pressures or duration of pylorospasm. Spearman (rho) correlation coefficient determined. N for each group shown to the right. $p<0.05$ considered significant.

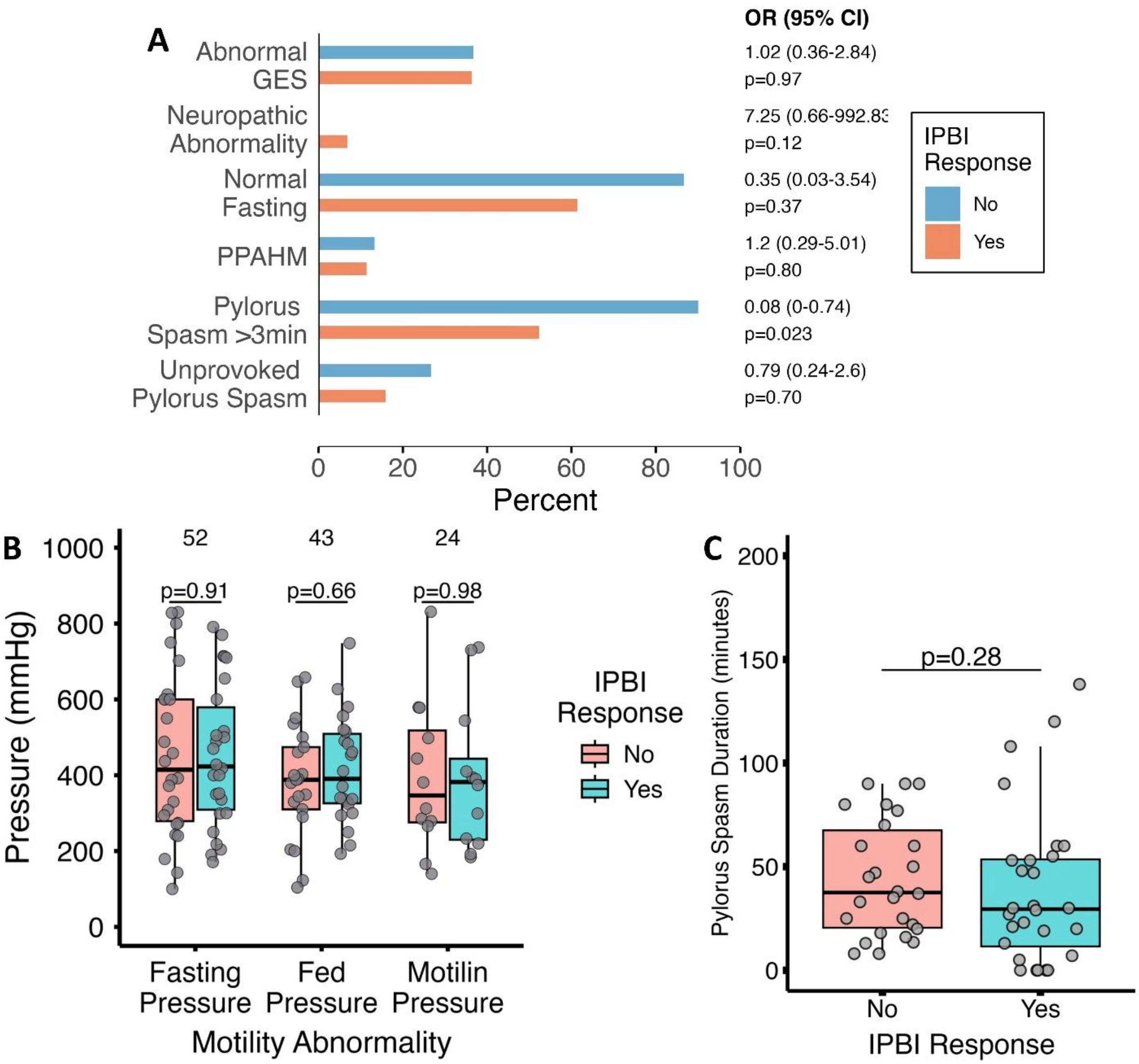

Supplemental Figure 4. Association of IPBI response with gastric motility abnormalities.
A) Reduced prolonged pylorus spasm in patients that responded to IPBI. Comparison by Binary logistic regression. **B-C)** No differences in ADM measures or Duration of Pylorospasm based on IPBI response. Comparison by Mann-Whitney. p<0.05 considered significant.

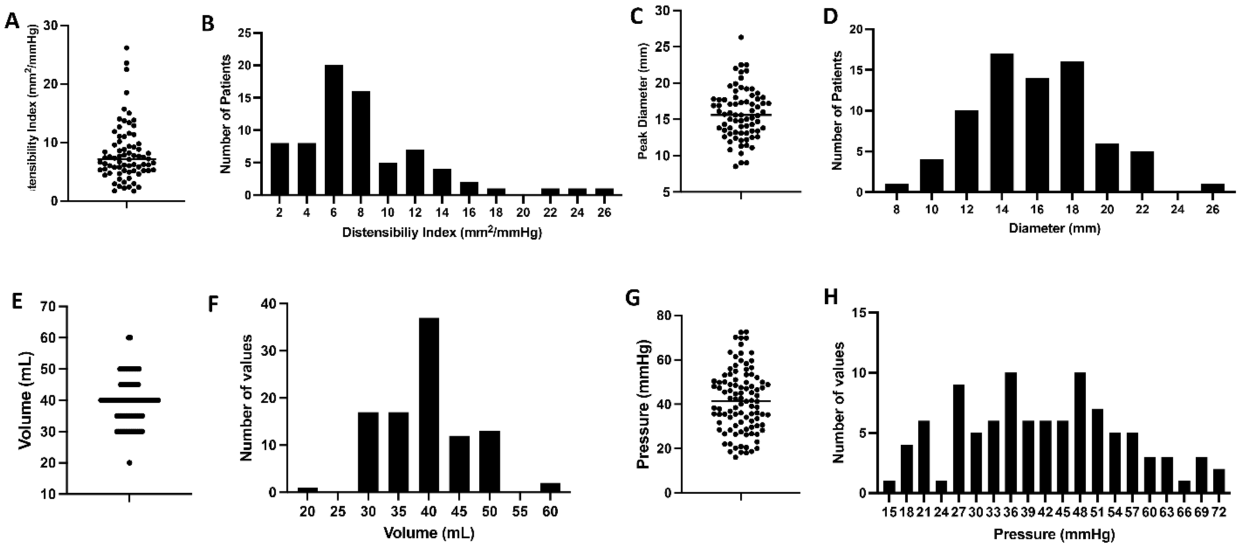

Supplemental Figure 5. Distribution of pyloric FLIP parameters. Non-normalized distribution observed for peak pyloric distensibility index (pDI) **(A-B)** with most values <10 mm2/mmHg. Normalized distribution of peak diameter **(C-D)**, and volume **(E-F)** and pressure **(G-H)** at the peak pDI.

Supplemental Table 1. Patient Characteristics Based on IPBI Response.

Characteristic[1]	Overall N=74	IPBI Non-Responsive N=30	IBPI Responsive N=44	p-value[2]
**Demographics**				
Age (years)	15.2±5.7	14.7±5.8	15.5±5.6	0.36
Gender (female)	58 (78)	23 (77)	35 (44)	0.99
Race/Ethnicity				
White	57 (77)	22 (73)	35 (80)	0.71
Black	5 (7)	3 (10)	2 (5)	
Asian	1 (1)	0 (0)	1 (2)	
Other	11 (15)	5 (17)	6 (14)	
Hispanic/Latinx	4 (5)	2 (7)	2 (5)	1
**Growth Parameters**				
Weight Z-score	-0.2±1.3	0.2±1.2	-0.6±1.2	0.008*
BMI Z-score	-0.1±1.2	0.4±1.3	-0.4±1.1	0.007*
**Symptoms Prior to IPBI[2]**				
Vomiting	36 (49)	14 (47)	22 (50)	0.96
Nausea	58 (78)	21 (70)	37 (84)	0.25
Abdominal Pain	63 (85)	27 (90)	36 (82)	0.51
Early Satiety	48 (65)	18 (60)	30 (68)	0.63
Distension/Bloating	53 (72)	22 (73)	31 (71)	0.99
Poor Weight Gain/Weight Loss	38 (51)	11 (37)	27 (61)	0.06
**Medical History[2]**				
Feeding Support	33 (44)	15 (50)	18 (41)	0.59
Cyclic Vomiting Syndrome	2 (3)	0 (0)	2 (5)	0.51
Chronic pain	52 (70)	20 (67)	32 (73)	0.76
Upper GI Series Performed	65 (88)	26 (87)	39 (89)	1
Abnormal GES[3]	23 (44)	9 (47)	14 (42)	0.96
Irritable Bowel Syndrome	29 (39)	13 (43)	16 (36)	0.72
Anxiety/Depression	46 (62)	21 (70)	25 (57)	0.37
Ehlers-Danlos/hypermobility	38 (51)	13 (43)	25 (57)	0.37
POTS/Orthostatic Intolerance	56 (76)	22 (73)	34 (77)	0.91
Abnormal biopsies[4]	15 (21)	8 (28)	7 (17)	0.45
**Pyloric FLIP[1]**				
FLIP measurements	3±1	3±1	3±1	0.90
Volume (mL)[5]	40±7	40±6	40±7	0.90
Diameter (mm)[6]	16±4	15±3	16±4	0.37
DI (mm^2/mmHg)[5]	8±5	7±4	9±5	0.008*
Pressure[5]	39±13	40±11	38±15	0.41

1. For continuous variable, display is mean±SD. For categorical variables, display is n (%)

2. Categorical variable: Chi-Square test, Normalized continuous variable: t-test, non-normalized continuous variable: Mann-Whitney. *p-value<0.05
3. 52 patients had 4-hour solid phase gastric emptying study (GES)
4. Only 70 of 74 patients received biopsies at time of pyloric FLIP. Abnormal biopsies included: Eosinophilic Esophagitis, ganglioneuroma, *H. pylori* gastritis, Intra-epithelial lymphocytes in the duodenum, peptic duodenitis, reflux esophagitis, reflux esophagitis + gastritis.
5. Measurements taken at peak distensibility index (DI)
6. Measurements taken at peak diameter
